# Concerns about the misleading conclusions regarding estimation of the net energy value of soybean meal relative to corn using the caloric efficiency approach in pigs

**DOI:** 10.1186/s40104-020-00519-1

**Published:** 2020-11-24

**Authors:** Shuai Zhang, Zhongchao Li, Ling Liu

**Affiliations:** State Key Laboratory of Animal Nutrition, Ministry of Agriculture and Rural Affairs Feed Industry Centre, China Agricultural University, Beijing, 100193 China

**Keywords:** Caloric efficiency, Corn, Net energy, Pig, Soybean meal

## Abstract

Apart from energy balance trials such as calorimetry, growth trials could also be used to estimate the energy values of feed ingredients with caloric efficiency as an indicator. Recent work used such methods reported greater net energy (NE) value of soybean meal (SBM) relative to corn in nursery pigs. We theoretically compared the NE values of SBM and corn according to the definition of NE and properties of the major chemical compositions in each ingredient. Meanwhile, we thoroughly examined the diet formulations and related analysis used in this work and compared this study with some peer works. We found that this study may suffer from problems with experimental design, reference citation, and data interpretation. In summary, the conclusion from the recent work that the SBM NE value may be greater than the corn NE value is likely to be erroneous.

## Main text

Net energy (NE) is the ideal basis to express both energy requirements of pigs and energy values of feeds [1]. Therefore, it is necessary to accurately evaluate the NE value of common feed ingredients when formulating cost-efficient diets based on their combinations. Calorimetry is the traditional approach to determine the NE value of diets and ingredients, but it relies on specialized equipment, and is a time-consuming and laborious work. Instead, more convenient approaches such as prediction equations based on chemical components of diets and ingredients can be used to estimate the NE values of feed ingredients rapidly.

In the July issue of *Journal of Animal Science and Biotechnology*, Cemin et al. [2] presented an approach of using caloric efficiency (CE) to estimate the NE value of soybean meal (SBM) relative to corn in nursery pigs. The authors reported improved CE in nursery pigs consuming diets formulated based on NE values from NRC [1] when the inclusion level of dietary SBM increased from 17.5% to 40% [2]. Therefore, Cemin et al. [2] concluded that NRC [1] underestimated the NE value of SBM, which ranges between 105% to 125% of corn energy. Although respecting the authors’ efforts, we have several concerns about data interpretation, experimental design, and the citation of related works, which might lead to misleading findings and conclusions. Specifically, we believe that the authors did not take the NE for lipid deposition into consideration when evaluating the NE value of SBM. Moreover, we found some errors in experimental diet formulation and when the authors cited some peer-reviewed works to support their inferences. Thus, the conclusion that the SBM NE value may be greater than the corn NE value is questionable.

Net energy is defined as metabolizable energy (ME) minus heat increment (HI) [1]. It has been widely accepted that the HI resulting from protein intake is much higher than that from carbohydrates and fat intake in mammals [3, 4]. When expressed as a percentage of ME, the HI of feeding carbohydrate and fat to mammals vary from 6% to 15% and 4% to 10%, respectively, while dietary protein can produce 30% HI in mammals and birds [3, 4]. In pigs, Le Bellego et al. [5] observed averaged 7 kJ decrease in heat production and averaged 3.5 kJ decrease in urine energy loss when replacing 1 g of protein with 1 g of starch in diets. In agreement, Noblet et al. [6] and van Milgen et al. [7] estimated the conversion efficiency of ME to NE for protein, starch, and fat to be 0.58, 0.82, and 0.90, and 0.52, 0.84, and 0.88, respectively. Considering the much higher crude protein content in SBM and much higher starch content in corn, the efficiency of ME utilization in SBM should be lower than that in corn. Coupled with the lower ME value in SBM [1], the NE value of SBM should be no greater than that of corn.

Net energy consumed from feed can divide into NE for maintenance (NE_m_), NE for protein deposition (NE_p_), and NE for lipid deposition (NE_l_) for pigs in growing-finishing stages [1]. As stated by Cemin et al. [2] in the article, the CE approach is more likely to be used to estimate the “productive energy” of a test ingredient, which could be on either digestible energy (DE), ME, or NE basis. The concept of CE relies on the measurement of the gain to feed ratio (G:F) without any adjustment for differences in chemical compositions of the body weight (BW) gain over the trial. This adjustment may be unnecessary if the composition of BW gain is not affected by the dietary treatments. However, in the present situation, chemical composition and especially the lipid content of BW gain must be greatly affected by the high inclusion level of SBM or the high crude protein content of the diet. Moreover, the potential of young piglets to deposit body protein is quite high, resulting in extra body protein gain and associated BW gain under any additional supply of balanced proteins. Besides, lipid tissues of young piglets still keep growing even though at relatively low rates, but making few contributions to the BW gain [8]. As a result, the energy content of BW gain should be quite different between dietary treatments in this case, and the calculation of CE without any adjustment is meaningless. This limitation of the CE approach is briefly mentioned by Cemin et al. [2] in the discussion, but no subsequent consequence in the interpretation of their results.

Regarding the experimental design, the control diet and growth stages of pigs may influence the energy values of the feed ingredients estimated by the CE approach, as stated by Boyd and Zier-Rush [9] in their early technical report of the CE trial, who used finishing pigs and the same control diet in all experiments. However, Cemin et al. [2] used different control diets in Exp. 1 and Exp. 2, with 21% and 17.5% SBM addition, respectively, leading to different results on growth performance and energy values of ingredients. We believe the post-hoc comparison is also needed to analyze the growth performance data among the treatment groups. With such analysis, there would be no difference in G:F among diets with 21%, 27%, and 39% SBM supplementation and the CE value between diets with 33% and 39% SBM supplementation in Exp. 1. Notwithstanding the lysine content was constant, the relatively high crude protein level in treatment groups such as 39% SBM addition could suppress the feed intake of pigs, leading to impaired G:F, which were shown in Exp. 1 but were not the situation in Exp. 2, and the authors did not give any explanation on such results. Moreover, the total rations of the ingredients in all diets are different, none of which are exactly equal to 100% (Table 1), and there are also some discrepancies between measured and calculated crude protein levels especially in Exp. 2 (Table 1), which we think may indicate erroneous SBM addition levels in actual diet formulation.

**Table 1:** The total rations of the ingredients and the discrepancy between calculated and measured crude protein levels in diets used by Cemin et al. [2]

Items	Treatment groups (inclusion level of soybean meal, %)					
**Exp. 1**	**21**	**27**	**33**	**39**		
Total ration, %	100.023	100.026	100.010	100.018		
Calculated crude protein level, %	19.2	21.3	23.4	25.6		
Measured crude protein level, %	20	21.4	24.2	25.9		
**Exp. 2**	**17.5**	**22.0**	**26.5**	**31.0**	**35.5**	**40.0**
Total ration, %	99.995	100.005	100.005	100.006	99.987	100.006
Calculated crude protein level, %	18.9	20.5	22.1	23.7	25.3	26.9
Measured crude protein level, %	17.2	19.2	20.2	22.7	23.7	25.6

Furthermore, Cemin et al. [2] referred to the NE value of SBM measured by Li et al. [10] using indirect calorimetry to support their inferences. Nevertheless, we believe the authors made a mistake when made the comparison, because the SBM NE value of 2709 kcal/kg reported by Li et al. [10] was on a dry matter basis, while the corn NE value of 2672 kcal/kg listed in NRC [1] estimated by prediction equation was on an as-fed basis, meaning that we cannot compare those results directly. Although the SBM NE value measured using indirect calorimetry was greater than that using the prediction equation, it is always lower than the corn NE value when compared in the same system — either in NRC [1] or reports from Li et al. [10] (Table 2).

**Table 2:** The net energy values of corn and soybean meal from NRC [1] and from Li et al. [10] in both dry matter basis and as-fed basis

Items	Dry matter basis	As-fed basis
Net energy value of corn, kcal/kg		
From NRC [1]	3026	2672
From Li et al. [10]	2978	2604
Net energy value of soybean meal^a^, kcal/kg		
From NRC [1]	2419	2148
From Li et al. [10]	2709	2403

^a^The crude protein level is 43%

Finally, previous peer works also used growth trials and young piglets to validate the NE value of corn and SBM from NRC [1]. According to Boyd et al. [11], pigs with initial BW of 12.7 kg and end BW of 30.8 kg were fed diets formulated with corn and SBM using NE value from NRC [1], and the inclusion levels of SBM were 25.9%, 29.3%, 32.4%, 35.6% and 38.8%, similar to those set in Exp. 1 conducted by Cemin et al. [2]. However, Boyd et al. [11] observed constant G:F among the five treatment groups (0.65, 0.66, 0.65, 0.65, and 0.65, ± 0.01, *P* = 0.4769), and indicated that the SBM NE value is 0.80 times as the corn NE value, further proved that corn could provide more NE value than SBM in growing-finishing pigs.

Overall, the study by Cemin et al. [2] suffers from some problems in experimental design, reference citation, and data interpretation, and their conclusion about the relationship between SBM NE value and corn NE value is likely to be erroneous. We believe that the NE values determined by the direct experimental approaches, such as indirect calorimetry, or by the prediction equations, are more reliable.

## Data Availability

Not applicable.

## Abbreviations

BW: Body weight
CE: Caloric efficiency
DE: Digestible energy
G:F: Gain to feed ratio
HI: Heat increment
ME: Metabolizable energy
NE: Net energy
SBM: Soybean meal
